# User-Initiated Symptom Assessment With an Electronic Symptom Checker: Protocol for a Mixed Methods Validation Study

**DOI:** 10.2196/41423

**Published:** 2023-07-19

**Authors:** Ville Liu, Tuomas H Koskela, Minna Kaila

**Affiliations:** 1 Faculty of Medicine University of Helsinki Helsinki Finland; 2 Faculty of Medicine and Health Technology Tampere University Tampere Finland; 3 Center of General Practice Tampere University Hospital Tampere Finland; 4 Medical Society Duodecim Suomalainen Lääkäriseura Duodecimry Helsinki Finland

**Keywords:** triage, symptom assessment, self-care, health service accessibility, telemedicine, health service research, internet, validation study, primary health care, clinical studies, telehealth

## Abstract

**Background:**

The national Omaolo digital social welfare and health care service of Finland provides a symptom checker, Omaolo, which is a medical device (based on Duodecim Clinical Decision Support EBMEDS software) with a CE marking (risk class IIa), manufactured by the government-owned DigiFinland Oy. Users of this service can perform their triage by using the questions in the symptom checker. By completing the symptom checker, the user receives a recommendation for action and a service assessment with appropriate guidance regarding their health problems on the basis of a selected specific symptom in the symptom checker. This allows users to be provided with appropriate health care services, regardless of time and place.

**Objective:**

This study describes the protocol for the mixed methods validation process of the symptom checker available in Omaolo digital services.

**Methods:**

This is a mixed methods study using quantitative and qualitative methods, which will be part of the clinical validation process that takes place in primary health care centers in Finland. Each organization provides a space where the study and the nurse triage can be done in order to include an unscreened target population of users. The primary health care units provide walk-in model services, where no prior phone call or contact is required. For the validation of the Omaolo symptom checker, case vignettes will be incorporated to supplement the triage accuracy of rare and acute cases that cannot be tested extensively in real-life settings. Vignettes are produced from a variety of clinical sources, and they test the symptom checker in different triage levels by using 1 standardized patient case example.

**Results:**

This study plan underwent an ethics review by the regional permission, which was requested from each organization participating in the research, and an ethics committee statement was requested and granted from Pirkanmaa hospital district’s ethics committee, which is in accordance with the University of Tampere’s regulations. Of 964 clinical user–filled symptom checker assessments, 877 cases were fully completed with a triage result, and therefore, they met the requirements for clinical validation studies. The goal for sufficient data has been reached for most of the chief symptoms. Data collection was completed in September 2019, and the first feasibility and patient experience results were published by the end of 2020. Case vignettes have been identified and are to be completed before further testing the symptom checker. The analysis and reporting are estimated to be finalized in 2024.

**Conclusions:**

The primary goals of this multimethod electronic symptom checker study are to assess safety and to provide crucial information regarding the accuracy and usability of the Omaolo electronic symptom checker. To our knowledge, this will be the first study to include real-life clinical cases along with case vignettes.

**International Registered Report Identifier (IRRID):**

DERR1-10.2196/41423

## Introduction

### Background

Seeking information online regarding medical symptoms is a common and well-known phenomenon worldwide [1-4]. According to a study in the United States, more than one-third of the surveyed adults regularly used the internet for self-diagnosis [5]. In another study, on average, 15% of the people queried on general-purpose search engines about symptoms associated with their conditions before receiving a proper medical diagnosis [6]. However, self-diagnostic web-based sources may be of varying quality, with misleading information and possibly false advertising [7,8].

To address these problems, health care digital applications have been introduced online, including self-diagnosing tools and symptom checkers. Based on users’ input of their symptoms, applications such as Babylon GP at Hand, K Health, Isabel, Symcat, Everyday Health, Ada, and WebMD use algorithms to help identify the relevant medical condition. An evaluation study of GP at Hand found that healthier individuals use e-services more than others [9]. Studies have also found that younger, highly educated people, and those with a higher socioeconomic status use eHealth services more actively [10-12]. Evidence on user behavior when using symptom checkers indicates that 3 in 4 users in a sample of nearly 500 users comply and follow electronic symptom checker recommendations, regardless of the urgency level [13]. One study [14] found that users are overcautious in deciding whether they require medical care at all and they miss identifying a considerable portion of emergencies. Further, women may be more risk averse than men in both types of decisions. However, users have mostly been satisfied with the electronic symptom checkers they use [10,15-18].

As with clinical decision-making in general, symptom checker questionnaires and algorithms used are based on research evidence, probabilities, and expert opinions as to whether the condition described is mild and self-limiting or whether it requires the intervention of a health care professional. In terms of urgency, an assessment is made on how soon the condition would worsen without treatment. For an accurate diagnosis, the user provides information that is required, complemented by clinical examinations, various diagnostic tests, and potential consultations with other medical personnel [19,20].

Previous studies have evaluated the accuracy of electronic symptom checkers for diagnosis and triage by using clinical case vignettes [21-30]. Vignettes appear to be a valid and comprehensive method that directly focuses on the process of care provided in actual clinical practice [31]. Under experimental conditions, the diagnostic accuracy of clinicians has been shown to be superior to that of electronic symptom checker tools in both primary and specialized health care [21-23].

Studies have shown that there are risks and room for error in digital triage. Users diagnosed with electronic symptom checkers may be referred to self-care even if they need professional help, and users for whom self-care would be sufficient are referred to professionals [21-23]. Specifically, self-care guidance should be limited to situations where it is safe and appropriate. A study to the point used data on more than 150,000 patient interactions with a web-based triage tool and found that the urgency of patients’ intended level of care decreased in more than 1 in 4 of the cases and increased in 1 in 20 cases, with the remaining patients remaining at the same level [32].

When comparing physicians’ and computers’ diagnostic accuracy by using case vignettes, physicians listed the correct diagnosis first more often than symptom checkers (72.1% vs 34%, respectively) [22]. There is limited evidence of patient safety hazards associated with the use of electronic symptom checkers [10,22,23,33]. However, a study comparing artificial intelligence and human doctors for the purpose of triage and diagnosis found that the artificial intelligence system was able to provide users triage and diagnostic information with a level of clinical accuracy and safety comparable to that of human comparators [34,35]. Electronic symptom checkers are, on average, more sensitive at recommending more urgent care than is actually needed [21,23,29,36]. Triage performance of symptom checkers has, on average, not improved over the course of 5 years [30].

In clinical practice, triage assessment and guidance are usually performed by health care professionals either during telephone contact or face-to-face, for example, at a health care center. In England, there were 1,678,880 calls made to National Health Service 111 in March 2021. Out of these calls triaged, 12% were referred to ambulance service, 11% to accident and emergency, 54% were recommended primary care, 8% advised to attend another service, and 14% were deemed not to need another service, that is, suitable for self-care [37].

Triage takes a lot of professional time and is of varying quality. In health care organizations, therefore, the goal is to unify triage and digitalize it with the help of symptom checkers. This is expected to increase service uniformity and efficiency as well as free up working hours [10,38].

### Study Objectives and Outcomes

The goal of this study was to ensure that the symptom checker is sensitive enough to recommend urgent care when it is needed and that it is specific enough to avoid unnecessary use of health care services. This is a mixed methods validation study of the Omaolo symptom checker (Table 1, Table 2, and Textbox 1). The focus of this study is on the usability of this tool, reliability of the assessments, and user safety [37-41]. The first part consists of comparing the Omaolo symptom checker assessment with an assessment made by an experienced primary care triage nurse to see how well they match. The second part complements the first by using patient case vignettes for the validation of the Omaolo symptom checker.

**Table 1:** Overview of the Omaolo symptom checker studies.

Characteristics	Substudy 1	Substudy 2
Study type	Quantitative clinical validation study	Validation with case vignettes study
Study objectives	Differences and similarities between the nurse’s and symptom checker’s (n=15) triage in real-life settings	To test if recommendations are safe on rare and acute cases, which could not be tested extensively in a real-life setting Case vignette accuracy is determined by comparing case vignette–filled symptom checker surveys with expert panel’s assessment (gold standard)
Study design and setting	Comparison of nurse’s triage to symptom checker’s triage	Comparison of symptom checker’s triage to triage nurse, general practitioner, and gold standard
Outcome	Triage frequencies, proportions of matches, symptom checker safety, specificity, and sensitivity	Case vignettes to complement the real-life assessment of the symptom checker

**Table 2:** Research questions for the clinical and case vignettes validation study.

Substudy	Research question
Clinical validation study: differences between the nurse’s and symptom checker’s triage in real-life settings	Are the recommendations of the symptom checker safe to use? In what proportion (% and 95% CIs for an estimate) of the assessments do the experienced nurse’s triage and the recommendation of action given by the symptom checker correspond or differ before the nurse has seen the recommendation of action given by the symptom checker? Are the recommendations of the symptom checker specific and sensitive enough to prevent unnecessary use of primary health care services? Does an experienced nurse change his or her assessment after seeing the recommendation for action by the symptom checker? What are the key factors that lead an experienced nurse’s triage to differ from the symptom checker’s recommendation? How many cases are there and to which symptom checker do they relate to wherein a triage nurse would refer the user to a heavier service than that recommended by the symptom checker (2 levels lighter is considered as unsafe recommendation for a symptom checker)? How many assessments can be found and to which symptom checker they relate to wherein the triage nurse would refer the user to a lighter service than that recommended by the symptom checker (2 levels heavier is considered as overconservative recommendation for a symptom checker)? Analysis of the content for the reasons given by the participants that influenced their triage when the individual assessments differed
Validation with case vignettes study: performance between nurses’ and symptom checker’s triage in case vignettes compared to expert panel assessment (gold standard)	In what proportion (% and 95% CIs for an estimate) of the assessments do the experienced nurse’s triage and the recommendation of action given by the symptom checker correspond to or differ from case vignette’s predetermined (assesses by expert panel) triage? Does an experienced nurse change his or her assessment after seeing the recommendation for action by the symptom checker? How many cases are there and to which symptom checker do they relate to wherein a triage nurse would refer the user to a heavier service than that recommended by the symptom checker (2 levels lighter is considered as unsafe recommendation for a symptom checker)? How many assessments can be found and to which symptom checker they relate to wherein the triage nurse would refer the user to a lighter service than that recommended by the symptom checker (2 levels heavier is considered as overconservative recommendation for a symptom checker)? Analysis of the content for the reasons given by the participants that influenced their triage when the individual assessments differed Sensitivity, specificity, positive and negative predictive values of the symptom checker and nurse assessments compared to expert panel assessments

**Questions presented in the research forms specifically for triage nurses.**

**Questions**

- How old are you?
- How much work experience do you have in assessing the need for care (triage) ? ____ years
- How did the user arrive at the reception? (walk-in or via telephone contact)
- Did you consult a doctor to assess the user’s need for treatment (triage)?
- Choose where the user should be referred according to the terms of the electronic symptom recommendation action guide (based on your own evaluation, triage)
- What was the most significant thing (observation, symptom, or discovery) that influenced your decision-making?
- Do you feel the need to change your assessment of need for treatment (triage) after seeing the responses and recommendation in the electronic symptom checker? (Yes/No)
- If yes, then why did you change the assessment of the need for treatment (triage) after seeing the responses and recommendation in the electronic symptom checker?
- If you feel it is necessary to change your triage assessment, reselect where the user should be referred to according to the classification terms of the electronic symptom checker’s recommendation.

### Intervention

#### Development of the Omaolo Symptom Checker

The clinical validation process of the Omaolo symptom checker proceeded at a national level and it complies with the European Union and Finnish medical device requirements with a CE marking (risk class: IIa) [42-45]. The Omaolo management team selected the topics to be included in the symptom checker on the basis of the most popular searches made on the evidence-based medicine guidelines (decision support for health care professionals by Duodecim Medical Publications Ltd), considering the most common reasons for visiting health centers and joint emergency services [46]. The Omaolo symptom checker is intended for users older than 15 years. There is no upper age limit, except for the symptom checker for urinary tract infections, which is intended for women aged 18-65 years.

The reasoning of each symptom assessment consists of 1 algorithm, which uses a common function library as an aid. All traffic goes through the engine of the evidence-based medicine decision support device, the application programming interface and related filters and conversions, and data processing. However, the algorithm that has the most central effect on reasoning can be separated from each individual symptom assessment within the symptom checker.

#### Description of the Omaolo Symptom Checker

Omaolo is a national web-based service for social welfare and health care. The purpose of Omaolo is to promote the health and well-being of citizens. Omaolo supports self-care and helps people to contact public health care professionals, if necessary. Omaolo is a medical device with a CE marking, manufactured by government-owned DigiFinland Oy founded in the year 2017. Omaolo meets the requirements set for medical devices.

The aim of the Omaolo symptom checker (Textbox 2) is to identify, based on the assessment of alarm symptoms and other pre-existing conditions, situations that require immediate or urgent assessment and to conduct follow-up examinations and treatment without delay in situations where conservative treatment may lead to complications. Both the questionnaire for the user and the algorithm that the symptom checker uses are based on research evidence (Multimedia Appendix 1), especially on the need to treat different combinations of symptoms and in other respects on medical experience (Textbox 2) [42-50].

**List of symptoms selected for the Omaolo symptom checker (n=15) and sources of medical knowledge and regulations applied in creating them [42-50] (full detailed references are listed in Multimedia Appendix 1).**

**Omaolo symptom checker**

- Anal region symptom
- Cough
- Diarrhea
- Discharge from the eye, watery or reddish eye
- Headache
- Heartburn
- Knee symptom or injury
- Lower back pain or injury
- Oral health
- Painful or blocked ear
- Respiratory tract infection
- Sexually transmitted disease
- Shoulder pain, stiffness, or injury
- Sore throat or throat symptom
- Urinary tract infection

**Regulations**

- Regulation (European Union) 2017/745 of the European Parliament and of the Council of April 5, 2017, on medical devices [42]
- Finnish Health Care Equipment and Supplies Act [43]
- New requirements and operational implications of medical devices (Finnish) [44]

**Sources of medical knowledge**

- Cochrane Database of Systematic Reviews [45]
- Articles in the DynaMed Plus; database produced by the EBSCO (Elton B Stephens CO) community [46]
- Finnish administrative sector of the Ministry of Social Affairs and Health publication “Basics of Emergency Care” [47]
- Finnish Current Care Guidelines [48]
- Evidence-Based Medicine Guidelines (Duodecim Medical Publications Ltd) [49]
- City of Helsinki Assessment of the Need for Urgent Care in Adult Patients (2014) [50]
- Preliminary studies investigating the likelihood of different combinations of symptoms and associated conditions requiring treatment

The symptom checker can only assess symptoms based on the information provided by the user. As with clinical decision-making in general, assessments of the need for treatment are based on the likelihood that the condition described is mild and self-limiting or that action by a professionally trained health professional is likely to be required to treat or prevent its worsening. In terms of urgency, the available information is used to assess whether and how quickly the condition is likely to worsen without treatment.

The symptom checker makes a general assessment and then recommends a course of action based on medical knowledge. It gives the user an idea of the quality and urgency of the treatment (triage) likely required. The user will be recommended to either treat their symptoms and their health problems themselves or be prompted to contact a health care professional who can provide a professional assessment of the situation and appropriate treatment, if necessary [39-41].

**Figure 1:**
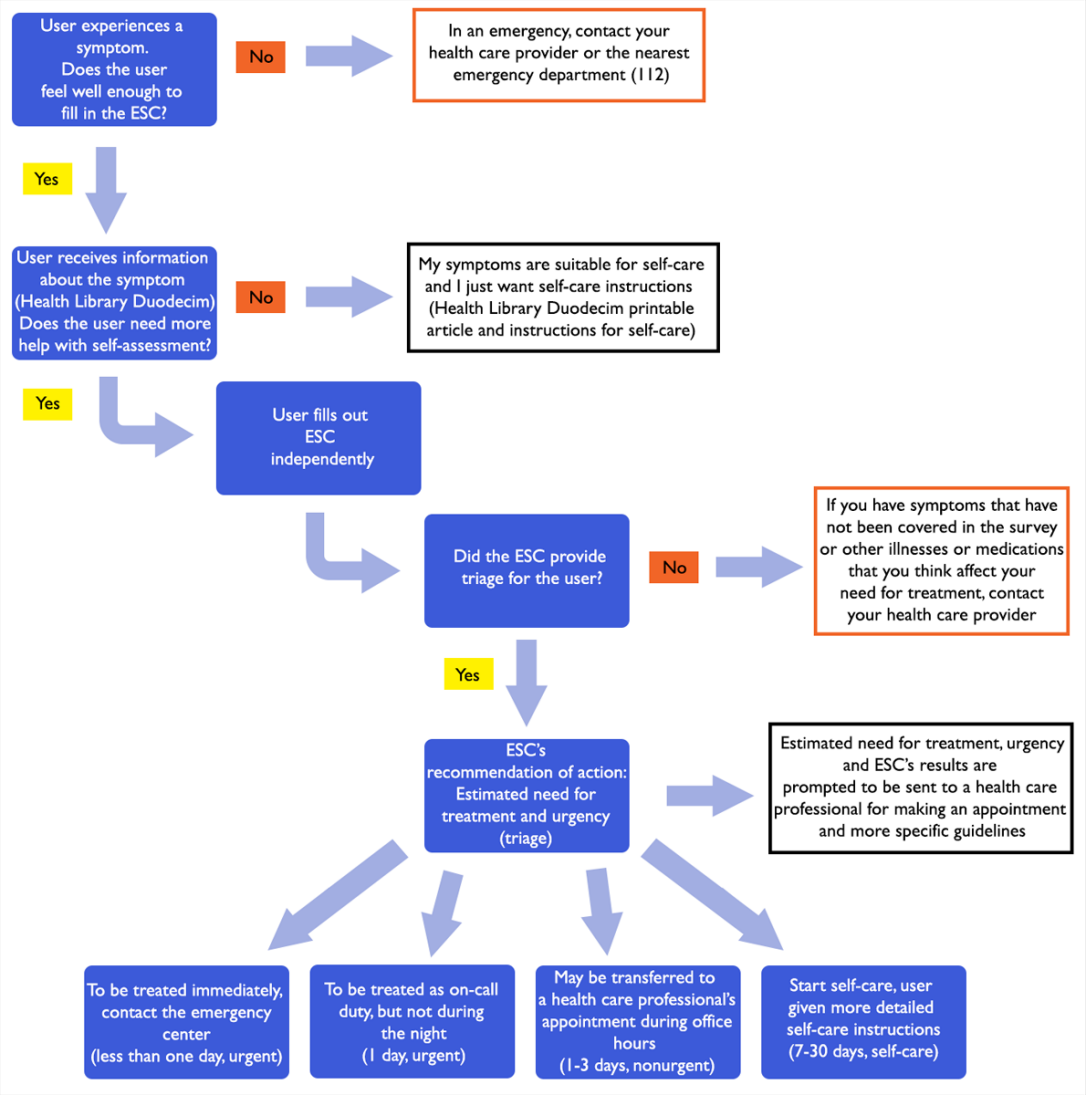
Flowchart for using Omaolo services and the decision tree of the symptom checker. ESC: electronic symptom checker.

The use of the Omaolo symptom checker is intended to be as simple as possible (Figure 1): the user initially receives reliable information about the symptom (articles in the Health Library Duodecim) with a short summary, helping the user in assessing their condition. If, however, the user is still unable to decide on the treatment, they can next answer the relevant symptom checker questions. These will, in turn, result in a recommendation by the checker on course of action and urgency. The checker does not result in a diagnosis.

The first priority of the symptom checker is to seek to identify any alarming symptoms that should prompt immediate contact to an emergency department. The aim is then to identify situations where a professional assessment is necessary and to determine the urgency of the assessment. For example, these include situations where antibiotic treatment may be necessary or situations where the symptom of the infection can be associated with an exacerbation of another disease. The combinations of symptoms are used to draw conclusions about the likely nature of the situation and to provide an appropriate recommendation. The successfully answered questions are saved, and the user can have it sent to a health care professional through the Omaolo symptom checker [39-41].

The user is encouraged to access the Duodecim Medical Library articles on the subject, which open as links in the questionnaire introductory text and feedback, since not all possible real-life situations can be covered. The user is also encouraged to consider whether they have other symptoms not covered by the questions of the information provided. To help with this, the following is displayed to the user at the end of the query: “If you have symptoms that have not been covered in the survey or other illnesses or medications that you think affect your need for treatment, contact your health care provider or, in an emergency, the nearest emergency department” [39-41].

## Methods

### Inclusion Criteria

To be eligible for the study, users had to be older than 18 years and experience some of the symptoms listed in the Omaolo symptom checker when participating. There is no upper age limit, except for the symptom checker of urinary tract infection, which is intended for women aged 18-65 years. The experienced nurses had to have at least 2 years of triage experience.

### Exclusion Criteria

The only exclusion criterion was refusal of the user to participate in this study.

### Pilot Study

The purpose of this pilot study was to examine the functionality of the study design of the symptom checker validation. The pilot was conducted at the Espoonlahti Health Center in July 2018. The pilot complied with the research plan and was performed to test the feasibility of the study design and the questionnaires. The pilot was conducted by collecting symptom checker assessments of 50 users with several symptoms at the same time. More than 200 users (Table 3) were interviewed to participate in this study, but only 50 symptom checker assessments were completed. The pilot caused minor changes mainly to question the formulations of the data collection documents and details of collecting the data.

**Table 3:** Reported reasons for users not taking part in the pilot study.

Reason for not participating in the study	Users (n=234)
Skeptical toward digitalization and e-services	33
Polite refusal without further explanation	76
Already has a reserved appointment time	41
On a matter regarding a family member’s well-being	10
Severity of symptoms hinders participation	29
User’s experienced symptoms are not found in the symptom checker	35
Fear of losing their place when waiting in the queue to meet the nurse	10

### Study Design and Setting

**Figure 2:**
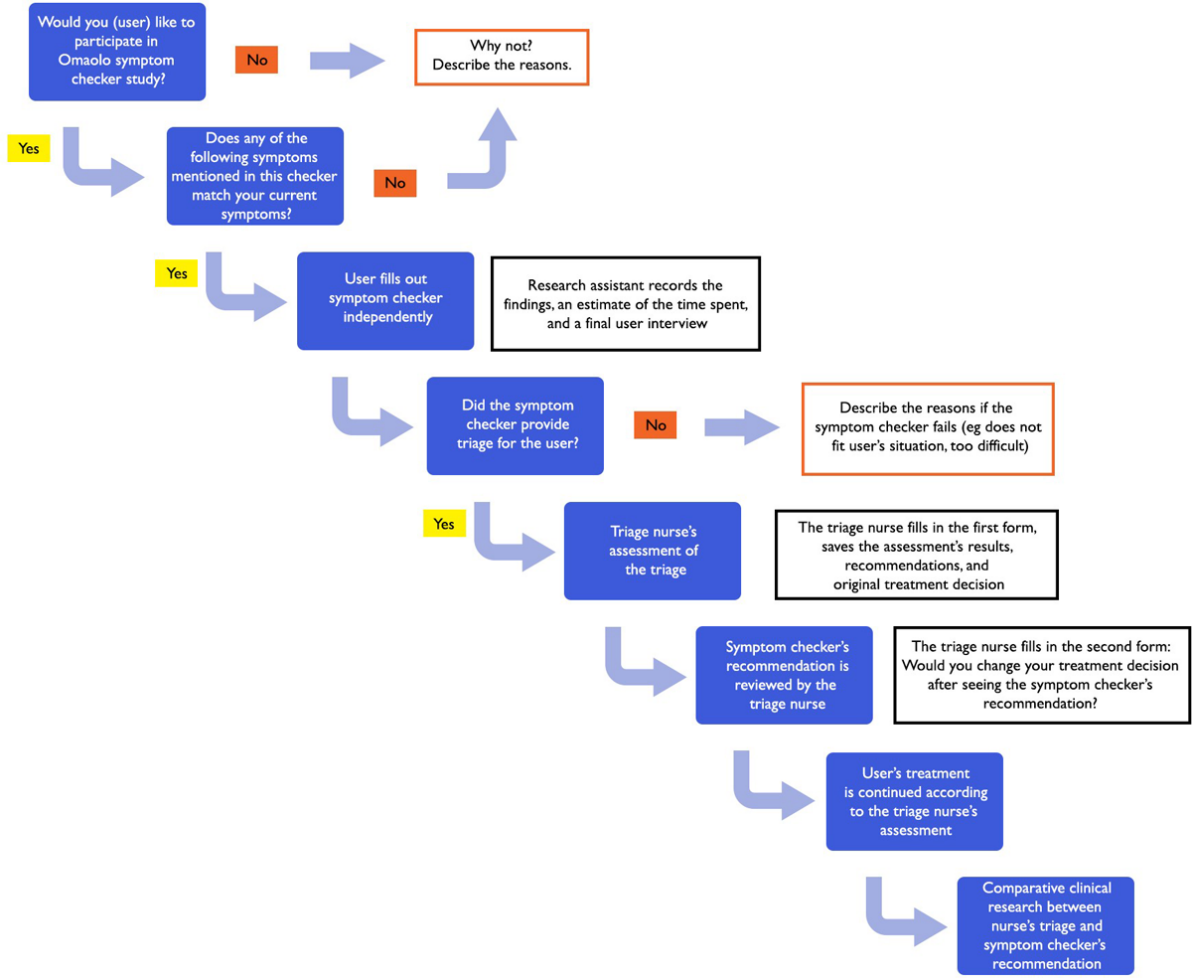
Study design for the clinical validation study.

The study setting is Finnish Primary Health Care, with 13 participating health care organizations nationwide. Each participating organization provided the study space where it is practically possible for the users to complete Omaolo symptom checker and triage by an experienced nurse. The organization also ensures that the target population of the study remains unscreened. Study users are recruited in units that use the walk-in model in primary health care settings, where clients come to a health center without prior contact (Figure 2).

**Figure 3:**
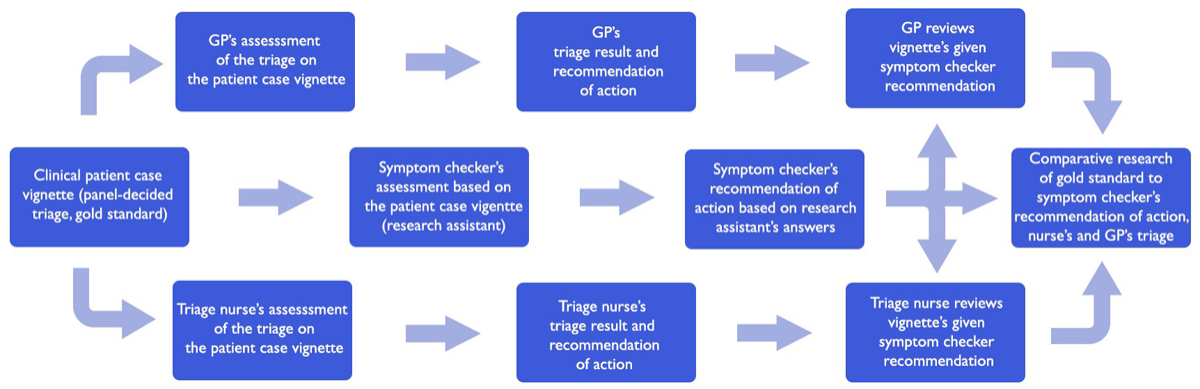
Study design for validation with case vignettes study. GP: general practitioner.

The validation with case vignettes (virtual clinical patient cases) will be used to complement the assessment of triage accuracy of some of the questions of the Omaolo symptom checker (Figure 3). Vignettes are either obtained from previous studies or produced for this study from a variety of clinical sources, including material used in medical education examinations. For example, an outcome that has been reached in a patient case in the educational environment is considered when creating a virtual patient case vignette [21-24]. Patient case vignettes are also produced from standardized clinical patient cases and panel-decided materials [22]. Panel-decided patient case vignettes and standardized patient case vignettes are a common method for developing and training health care professionals in their diagnostic ability to make treatment decisions [21].

The panel in our study aims to consist of 3-5 independent clinician general practitioners (GPs) and another 3-5 triage nurses. We will apply a modified Delphi procedure [51]. For the creation of clinical vignettes, the GPs are required to have over 5 years of primary care and emergency department experience. These GPs are not to be directly involved with Omaolo creation. In case of the vignette validation process, the panel first assesses the triage for case vignettes (gold standard). Second, the panel-decided triage is then compared with the nurse’s and GP’s triage and the symptom checker’s recommendation of action (this is possible in simulated cases where conditions are constant) (Figure 2). Case vignettes test the symptom checker and compare their performance with other providers in different triage levels by using standardized patient case examples [21-24]. Individual patient case vignettes are formed according to urgency classifications. Case vignettes also include common and rare diseases. The focus of this study will be on rare and acute cases, which cannot be tested extensively in real-life settings, for example, the number of cases collected for diarrhea and heartburn symptom checker is small (Table 4). Some of the vignettes will be derived from established sources [22].

**Table 4:** Data of the collected clinical user cases across the symptom checker.

Symptoms in Omaolo symptom checker	Approved assessments for clinical validation (n=877), n	Approved assessments for usability study (n=964), n
Anal region symptom	41	39
Cough	71	87
Diarrhea	21	23
Discharge from the eye, watery or reddish eye	65	64
Headache	41	45
Heartburn	24	19
Knee symptom or injury	55	56
Lower back pain or injury	65	69
Oral health	62	69
Painful or blocked ear	81	83
Respiratory tract infection	104	121
Sexually transmitted disease	39	30
Shoulder pain, stiffness, or injury	47	50
Sore throat or throat symptom	101	117
Urinary tract infection	60	60

### Sample Size Calculations

In a study performed by Gilbert et al [22], triage levels of 7 different symptom checker apps were tested and the mean percentage of safe advice provided by the apps was found to be 90.1% (SD 7.4%). Furthermore, a symptom checker app was found and defined to be safe to use if the particular app had safe advice performance within 1 SD compared to the GP’s (mean) percentage of given safe advice. The GP’s percentage of safe advice was 97% (SD 2.5%) [22]. For our study, hypothesis 1 (α) is that the Omaolo symptom checker is safe to use in the defined study settings (Table 1).

For the study group in the clinical validation study, we accept a safe performance of 97% when directly comparing symptom checker’s recommendation of action to nurse’s triage of the same user that has filled in the symptom checker successfully. We estimate the required sample size by assuming the given range of safe advice at 97% and by using a 95% confidence level, and we compute the confidence interval estimate for the true proportion of safe-filled symptom checker assessments (Figure 2). For each symptom, we aim to collect as many completed symptom assessments as possible.

For the study in validation with the case vignettes study, we assume that the Omaolo symptom checkers’ performance is similar to that of symptom checker apps defined as safe as described by Gilbert et al [22]. This includes that the suggested safe advice performance marginal and size of the difference between the study groups is acceptable (Figure 3).

### Data Analysis

The qualitative assessment of the study data from the open-ended questions of the survey was analyzed by qualitative inductive content analysis [52]. In this approach, the codes and themes derived from the data were suggested by the data rather than by a theoretical framework. The steps that we used for our analysis were iterative processes to familiarize ourselves with the qualitative data and to identify quotations with common concepts, code formation, grouping of codes into subthemes and themes, and creation of an explanatory thematic summary.

**Figure 4:**
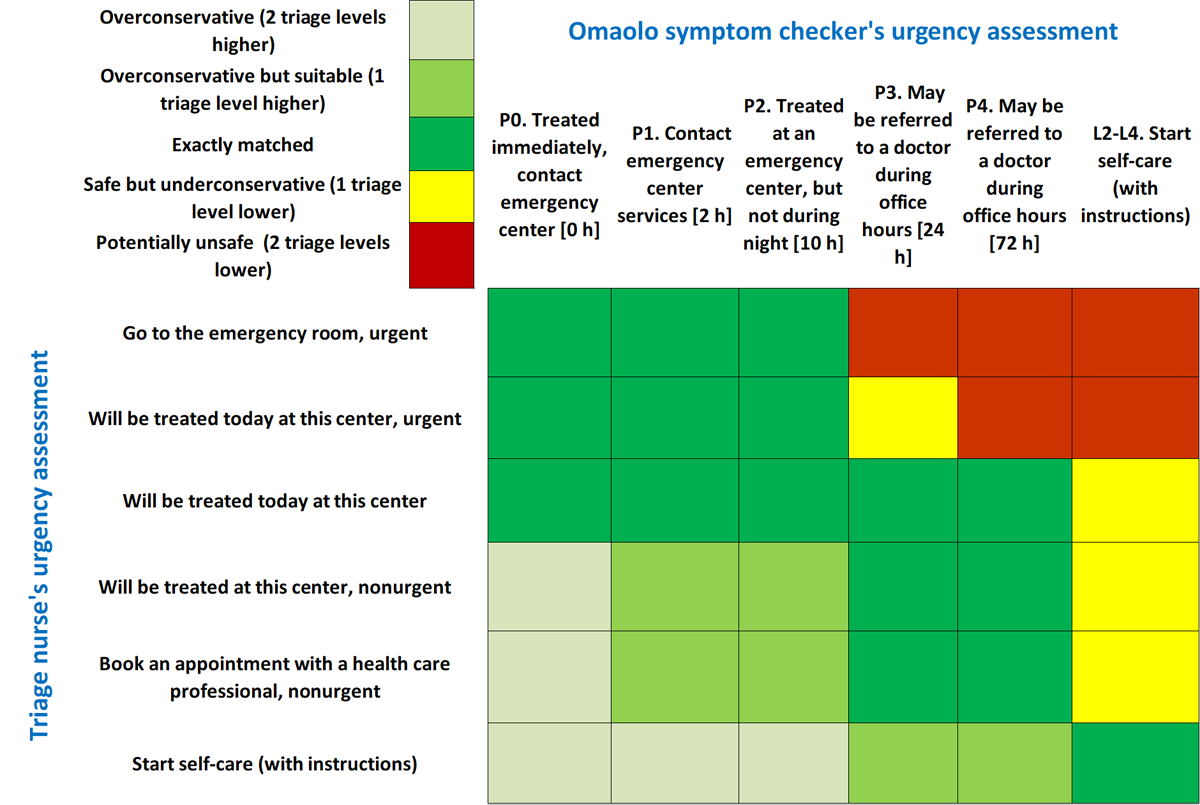
An example of a triage comparison chart with matched color coding differences in triage levels. Matching rows (triage nurse) to their respective columns (symptom checker) results in a safety assessment. P1-4, Finnish Ministry of Social Affairs and Health Classification of emergency care criteria; L2-4, referral urgency classes of Finnish Institute for Health and Welfare coding service.

The quantitative analysis in the clinical and vignette validation studies includes the results and findings of individual users, nurses, and study assistants being analyzed based on individually completed study forms. The results of the electronic symptom assessments compared to the assessment of an experienced nurse and case vignettes will be presented regarding each symptom checker individually. Possible differences in triage levels, including “overconservative,” “overconservative but suitable,” “exactly matched,” “safe but underconservative,” and “potentially unsafe,” for all symptom estimates will be reported (Figure 4) [22]. We will calculate the percentages of matches for each individual symptom checker and the 95% CIs.

A case is to be defined as safe if the conflict condition is not met and the recommendation for action given by the symptom assessment is at most 1 degree of urgency less urgent than the triage assessment of the same case. The definition of a conflict is as follows: cases assessed by the nurse as urgent/on-call duty but assessed by the electronic symptom checker as nonurgent/self-care.

### Ethics Approval

This study plan underwent an ethics review by the regional permission, which was requested from each organization participating in the research, and in addition to this, an ethics committee statement was requested and granted from Pirkanmaa hospital district’s ethics committee (ETL-Code:R18126), which is in accordance with the University of Tampere’s regulations. When recruiting participants for this study, the research assistant informs the user about the study, distributes the study information sheet, and then asks if the user is willing to participate in the study. If, after being informed, the user is willing to participate in the study, they are asked to sign a consent form in which the user acknowledges that they have received sufficient information about the study and agree to participate in it. The user is given an information sheet about the study, which contains information about the study and contact information in case the user wants to ask more about the study. The user was paid no amount of compensation. The user’s consent form is not connected to the response form with a personal identification code, that is, the users are anonymized. No medical record data are collected or combined with research forms. The users can withdraw their consent to the study at any time, but the completed forms cannot be destroyed after the data collection, because the consent form, which contains personal data, does not have an identification code that could be used to link the consent to other study forms. The most significant ethical issue related to the research setting is that the user’s participation in the research does not affect their chances of receiving timely treatment. In connection with the research protocol, all users who fill out the symptom checker will be forwarded to an appointment with an experienced nurse. Denial of treatment for users who refuse to participate in the study is strictly prohibited.

## Results

Data (Table 4) for this study were collected between June 1, 2018, and December 31, 2020, when users filled in electronic symptom checker questionnaires on arrival at the health centers (they also signed an informed consent form). The version of the Omaolo.fi symptom checker of 2019 was used. Few user cases were collected for diarrhea and heartburn in the clinical validation part of the study. In the supplementary case vignette validation study, the use of virtual clinical patients will be used to support the triage accuracy of chief symptom assessments with a small number of approved assessments for clinical validation (heartburn, diarrhea) in the Omaolo symptom checker. The focus will be on rare and acute cases, which cannot be tested extensively enough in real-life settings. Data collection was completed in September 2019, and the first feasibility and patient experience results were published by the end of 2020 [33]. Further results and publications are expected in 2023-2024. It is estimated that the analysis and reporting will be finalized during 2024.

## Discussion

### Principal Findings

This multidimensional study will be the first to assess both the usability and safety of the Omaolo symptom checker in the Finnish primary health care context. In addition, this research might provide information to help evaluate the reliability and possible shortcomings of this service. This information can then be used to support the further development of the service entity and thus improve the usability of the Omaolo service. The real benefit and safety of this service can be gauged from the written feedback of users who use the symptom checker in real clinical trial environments [53].

### Strengths and Weaknesses

The real-life setting is both a strength and a potential weakness of this study. A strength of this study is the large number of real-life users with real-life symptoms and real-life triage performed by a nurse. The first potential concern is the selection bias of the symptoms experienced when users fill the symptom checker questionnaires in health center waiting rooms, while in real-life settings, the symptom checker questionnaire is filled at home. The Omaolo symptom checker is designed for users older than 15 years, but in this study setting, only users older than 18 years are recruited. Second, the potential selection bias results from excluding users who are not able to complete the symptom checker questionnaire independently (inability to use the provided computer mouse or tablet devices). Further, users presenting with symptoms at the extremes of the spectrum may be problematic. Cases of mild self-care symptoms may be excluded, and users with serious acute symptoms may be rare in this setting. The symptom checker service does prompt users to contact health care services urgently when unable to fill in the questionnaire due to severe symptoms. The lack of urgent cases for some symptoms (eg, heartburn, diarrhea) in real-life settings is considered to be supplemented by virtual user cases, that is, case vignettes. The focus of validation with the case vignette study will be on rare and acute cases, which cannot be tested extensively enough in real-life settings. More common and less urgent situations can be covered even with the vignette study. Finally, although the production of research data was slowed down by the COVID-19 pandemic [54,55], the number of individual users of mobile medical apps and Omaolo.fi grew exponentially during the pandemic [56-58].

## Appendix

Multimedia Appendix 1 Sources of medical knowledge applied in creating the Omaolo electronic symptom checker.

## Abbreviations

GP: general practitioner

## Data Availability

The forms concerning individual users in the database are anonymized and cannot be combined with the user’s identification code to personal data. The information in the questionnaires is coded electronically and transferred to an electronic folder. The names or personal information of the users are not shown in this electronic file but only the user’s identification code. Paper forms with identification codes are stored in a locked cabinet. The consent form containing the user’s name and signature is kept in a separate locked cabinet. All paper forms are stored in the University of Tampere. The forms are disposed of with a shredder 10 years after the end of the study. The electronic data sets generated during and analyzed in this study are available from the corresponding author on reasonable request.
