# Supplementary material for: User-Initiated Symptom Assessment With an Electronic Symptom Checker: Protocol for a Mixed Methods Validation Study
Source: JMIR Res Protoc. 2023 Jul 19;12:e41423. doi: 10.2196/41423 (PMC10398552; doi:10.2196/41423)
Supplement: Multimedia Appendix 1 [file resprot_v12i1e41423_app1.docx]

Multimedia Appendix 1. Sources of medical knowledge applied in creating Omaolo ESC

Cochrane Database of Systematic Reviews [A1-A4]

[A1] Kenealy T, Arroll B. Antibiotics for the common cold and acute purulent rhinitis. Cochrane Database of Systematic Reviews 2013, Issue 6. Art. No.: CD000247. DOI: 10.1002/14651858.CD000247.pub3.

[A2] Reveiz L, Cardona AF. Antibiotics for acute laryngitis in adults. Cochrane Database of Systematic Reviews 2015, Issue 5. Art. No.: CD004783. DOI: 10.1002/14651858.CD004783.pub5.

[A3] Spinks A, Glasziou PP, Del Mar CB. Antibiotics for sore throat. Cochrane Database of Systematic Reviews 2013, Issue 11. Art. No.: CD000023. DOI: 10.1002/14651858.CD000023.pub4.

[A4] Smith SM, Fahey T, Smucny J, Becker LA. Antibiotics for acute bronchitis. Cochrane Database of Systematic Reviews 2014, Issue 3. Art. No.: CD000245. DOI: 10.1002/14651858.CD000245.pub3.

Articles in the DynaMed Plus - database produced by the EBSCO community [B1-B22]

[B1] DynaMed Plus [verkkojulkaisu]. Ipswich (MA): EBSCO Information Services. 1995 - . Record No. 116914, Gastroesophageal reflux disease (GERD); [Päivitys 11.4. 2016, luettu 31.12.2016]. Saatavilla http://www.dynamed.com/login.aspx?direct=true&site=DynaMed&id=116914.  Käyttöoikeus vaaditaan.

[B2] Dynamed Plus [verkkojulkaisu]. Streptococcal pharyngitis testing strategies. Saatavilla http://www.dynamed.com/topics/dmp~AN~T474264/Streptococcal-pharyngitis-testing-strategies, päivitys 25.3.2016.

[B3] DynaMed Plus [Internet]. Ipswich (MA): EBSCO Information Services. 1995-. Record No. 114958, Acute low back pain; (15.5.2017). <http://www.dynamed.com/topics/dmp~AN~T114958/Acute-low-back-pain>.

[B4] DynaMed Plus [Internet]. Ipswich (MA): EBSCO Information Services. 1995-. Record No. 11, Chronic low back pain; (30.6.2017). <http://www.dynamed.com/topics/dmp~AN~T116935/Chronic-low-back-pain>

[B5] DynaMed Plus [Internet]. Ipswich (MA): EBSCO Information Services. 1995-. Record No. 116345, Acute otitis media; (päivitys 6.12.2016, luettu 19.1.2017). Saatavilla https://www.dynamed.com/topics/dmp~AN~T116345/Acute-otitis-media-AOM. Edellyttää käyttöoikeutta.

[B6] DynaMed Plus [verkkojulkaisu]. Ipswich (MA): EBSCO Information Services. 1995-. Record No. 114773, Headache; (päivitys 24.6.2014, luettu 1.6.2016). Saatavilla [http://www.dynamed.com/login.aspx?direct=true&site=DynaMed&id=114773](http://www.dynamed.com/login.aspx?direct=true&site=DynaMed&id=114773). Edellyttää käyttöoikeutta.

[B7] DynaMed Plus [Internet]. Ipswich (MA): EBSCO Information Services. 1995-. Record No. 114537, Upper respiratory tract infection (URI) in adults and adolescents.  (päivitys 1.3.2017, luettu 6.3.2017). Saatavilla https://www.dynamed.com/topics/dmp~AN~T116345/Acute-otitis-media-AOM. Edellyttää käyttöoikeutta.

[B8] DynaMed Plus [verkkojulkaisu]. Ipswich (MA): EBSCO Information Services. 1995 -. Record No. 116345, Acute otitis media; (päivitys 16.5.2017, luettu 26.9.2017). Saatavilla https://www.dynamed.com/topics/dmp~AN~T116345/Acute-otitis-media-AOM. Edellyttää käyttöoikeutta.

[B9] DynaMed Plus [verkkojulkaisu]. Ipswich (MA): EBSCO Information Services. 1995-. Record No. 230359, Cerumen impaction; (päivitys 18.5.2017, luettu 26.9.2017). Saatavilla https://www.dynamed.com/topics/dmp~AN~T230359/Cerumen-impaction. Edellyttää käyttöoikeutta.

[B10] Dynamed Plus - Shoulder dislocation [verkkojulkaisu]  [http://www.dynamed.com/topics/dmp~AN~T115848/Shoulder-dislocation](http://www.dynamed.com/topics/dmp~AN~T115848/Shoulder-dislocation), päivitetty 9.7.2014.

[B11] Dynamed Plus- Adhesive capsulitis of shoulder [verkkojulkaisu]. <http://www.dynamed.com/topics/dmp~AN~T113935/Adhesive-capsulitis-of-shoulder>, päivitetty 22.12.2015.

[B12] Dynamed Plus -Impingement syndrome of rotator cuff [verkkojulkaisu] <http://www.dynamed.com/topics/dmp~AN~T114840/Impingement-syndrome-of-rotator-cuff>, päivitetty 20.5.2015.

[B13] Dynamed Plus -Recurrent subluxation of shoulder [verkkojulkaisu] <http://www.dynamed.com/topics/dmp~AN~T114357/Recurrent-subluxation-of-shoulder>, päivitetty 21.8.2014.

[B14] DynaMed Plus [verkkojulkaisu]. Ipswich (MA): EBSCO Information Services. 1995-. Record No. 116475, Hemorrhoids (päivitys 5.12.2016. Saatavilla http://www.dynamed.com/login.aspx?direct=true&site=DynaMed&id=116475. Edellyttää käyttöoikeutta.

[B15] DynaMed Plus [verkkojulkaisu]. Ipswich (MA): EBSCO Information Services. 1995-. Record No. 116635, Pruritus ani; (päivitys 13.6.2013). Saatavilla http://www.dynamed.com/login.aspx?direct=true&site=DynaMed&id=116635. Edellyttää käyttöoikeutta.

[B16] DynaMed Plus [verkkojulkaisu]. Ipswich (MA): EBSCO Information Services. 1995-. Record No. 113851, Anal fissure; (päivitys 2.6.2016). Saatavilla http://www.dynamed.com/login.aspx?direct=true&site=DynaMed&id=113851. Edellyttää käyttöoikeutta.

[B17] DynaMed Plus [verkkojulkaisu]. Ipswich (MA): EBSCO Information Services. 1995-. Record No. 113642, Colorectal cancer; (Päivitys 3.11.2016). Saatavilla http://www.dynamed.com/login.aspx?direct=true&site=DynaMed&id=113642.  Käyttöoikeus vaaditaan.

[B18] DynaMed Plus [verkkojulkaisu]. Ipswich (MA): EBSCO Information Services. 1995-. Record No. 116914, Gastroesophageal reflux disease (GERD); [Päivitys 11.4. 2016, luettu 31.12.2016]. Saatavilla http://www.dynamed.com/login.aspx?direct=true&site=DynaMed&id=116914.  Käyttöoikeus vaaditaan.

[B19] DynaMed Plus [verkkojulkaisu]. Ipswich (MA): EBSCO Information Services. 1995-. Record No. T90062; Päivitys 27.4.2017. Saatavilla http://www.dynamed.com/login.aspx?direct=true&site=DynaMed&id= T900062.  Käyttöoikeus vaaditaan.

[B20] DynaMed Plus [verkkojulkaisu]. Ipswich (MA): EBSCO Information Services. 1995-. Record No.116741, Infectious conjunctivitis; päivitys 15.7.2016. Saatavilla https://www.dynamed.com/topics/dmp~AN~T116345/Acute-otitis-media-AOM. Edellyttää käyttöoikeutta.

[B21] DynaMed Plus [verkkojulkaisu]. Ipswich (MA): EBSCO Information Services. 1995-. Record No.115480, Allergic conjunctivitis; päivitys 22.6.2016. Saatavilla https://www.dynamed.com/topics/dmp~AN~T116345/Acute-otitis-media-AOM. Edellyttää käyttöoikeutta.

[B22] DynaMed Plus  [verkkojulkaisu]. Ipswich (MA): EBSCO Information Services. 1995-. Record No. 113814, Acute Bronchitis; päivitys 26.09.2017. Saatavilla http://www.dynamed.com/topics/dmp~AN~T113814. Edellyttää käyttöoikeutta.

Finnish Current Care Guidelines [C1-C13]

[C1] Itselääkitys  [verkkojulkaisu]. Suomalaisen Lääkäriseuran Duodecimin ja Suomen Apteekkariliiton asettama työryhmä. Helsinki: Suomalainen Lääkäriseura Duodecim, 2016 (luettu 9.8.2016).  Saatavilla: www.kaypahoito.fi

[C2] Alahengitysinfektiot (aikuiset)  [verkkojulkaisu]. Suomalaisen Lääkäriseuran Duodecimin, Suomen Keuhkolääkäriyhdistyksen, Suomen Infektiolääkärit ry:n ja Suomen Yleislääketieteen Yhdistys ry:n asettama työryhmä. Helsinki: Suomalainen Lääkäriseura Duodecim, 2015 (viitattu 9.8.2016) Saatavilla www.kaypahoito.fi

[C3] Sivuontelotulehdus  [verkkojulkaisu]. Suomalaisen Lääkäriseuran Duodecimin ja Korva-, nenä- ja kurkkutaudit sekä Pään ja kaulan kirurgia ry:n asettama työryhmä. Helsinki: Suomalainen Lääkäriseura Duodecim, 2013.  Saatavilla Internetissä: www.kaypahoito.fi

[C4] Virtsatieinfektiot [verkkojulkaisu]. Suomalaisen Lääkäriseuran Duodecimin, Suomen Nefrologiyhdistys ry:n, Kliiniset mikrobiologit ry:n, Suomen Infektiolääkärit ry:n, Suomen Kliinisen Kemian Erikoislääkäriyhdistys ry:n, Suomen Lastenlääkäriyhdistys ry:n, Suomen Urologiyhdistyksen ja Suomen yleislääketieteen yhdistys ry:n asettama työryhmä. Helsinki: Suomalainen Lääkäriseura Duodecim, 2015 (viitattu 31.10.2017). Saatavilla www.kaypahoito.fi

[C5] Sivuontelotulehdus (online). Käypä hoito -suositus. Suomalaisen Lääkäriseuran Duodecimin ja Korva-, nenä- ja kurkkutaudit - Pään ja kaulan kirurgia ry:n asettama työryhmä. Helsinki: Suomalainen Lääkäriseura Duodecim, 2013.  Saatavilla Internetissä: [www.käypähoito.fi](http://www.käypähoito.fi)

[C6] Alaselkäkipu. Suomalaisen Lääkäriseuran Duodecimin ja Suomen Fysiatriyhdistyksen asettama työryhmä. Helsinki: Suomalainen Lääkäriseura Duodecim, 2017.  Saatavilla Internetissä: [www.kaypahoito.fi](http://www.kaypahoito.fi)

[C7] Alahengitystieinfektiot (aikuiset) (online). Käypä hoito -suositus. Suomalaisen Lääkäriseuran Duodecimin, Suomen Keuhkolääkäriyhdistyksen, Suomen Infektiolääkärit ry:n ja Suomen Yleislääketieteen Yhdistys ry:n asettama työryhmä. Duodecim, 2015 (viitattu 25.2.2017).

[C8] Nielutulehdus (online). Käypä hoito -suositus. Suomalaisen Lääkäriseuran Duodecimin, Suomen Yleislääketieteen yhdistyksen, Suomen Otolaryngologiyhdistyksen, Suomen Infektiolääkärit ry:n ja Kliiniset mikrobiologit ry:n asettama työryhmä. Helsinki: Suomalainen Lääkäriseura Duodecim, 2013.  Saatavilla Internetissä: www.käypähoito.fi

[C9] Ylävatsavaivaisen potilaan tutkiminen ja hoito [verkkojulkaisu]. Käypä hoito -suositus. Suomalaisen Lääkäriseuran Duodecimin ja Suomen Gastroenterologiayhdistyksen asettama työryhmä. Helsinki: Suomalainen Lääkäriseura Duodecim, 2013 (viitattu 9.4.2017).  Saatavilla: [www.käypähoito.fi](http://www.käypähoito.fi)

[C10] Käypä hoito - Olkapään jännevammat [verkkojulkaisu]. [http://www.kaypahoito.fi/web/kh/suositukset/suositus;jsessionid=D16DA5FFAB986E80763C2FB3A9378960?id=hoi50099](http://www.kaypahoito.fi/web/kh/suositukset/suositus;jsessionid=D16DA5FFAB986E80763C2FB3A9378960?id=hoi50099), julkaistu 23.11.2014

[C11] Polvi- ja lonkkanivelrikko [verkkojulkaisu]. Käypä hoito -suositus. Suomalaisen Lääkäriseuran Duodecimin ja Suomen Ortopediyhdistys ry:n asettama työryhmä. Helsinki: Suomalainen Lääkäriseura Duodecim, 2014.  Saatavilla: [www.käypähoito.fi](http://www.xn--kyphoito-0zac.fi/)

[C12] Migreeni [verkkojulkaisu]. Suomalaisen Lääkäriseuran Duodecimin ja Suomen Neurologinen yhdistys ry:n asettama työryhmä. Helsinki: Suomalainen Lääkäriseura Duodecim, 2015 (viitattu 16.2.2017). Saatavilla internetissä: www.kaypahoito.fi

[C13] Päänsärky (lapset). Käypä hoito -suositus [verkkojulkaisu]. Suomalaisen Lääkäriseuran Duodecimin, Suomen Lastenneurologinen Yhdistys ry:n asettama työryhmä. Helsinki: Suomalainen Lääkäriseura Duodecim, 2015 (viitattu 1.6.2016).  Saatavilla Internetissä: www.kaypahoito.fi.

Evidence-Based Medicine Guidelines (Duodecim Medical Publications Ltd.) [D1-D44]

[D1] Knuuttila A. Veriyskä. Lääkärin tietokannat/Lääkärin käsikirja  [verkkojulkaisu]. Helsinki: Kustannus Oy Duodecim, päivitys 15.6.2016.

[D2] Knuuttila A. Keuhkosyöpä. Lääkärin tietokannat/Lääkärin käsikirja  [verkkojulkaisu]. Helsinki: Kustannus Oy Duodecim, päivitys 15.6.2016.

[D3] Sidekalvontulehdus (toimitus). Lääkärin tietokannat/Lääkärin käsikirja [verkkojulkaisu]. Helsinki: Kustannus Oy Duodecim, päivitys 12.9.2016.

[D4] Kari, O. Piilolasien käyttäjän kipeä silmä. Lääkärin tietokannat/Lääkärin käsikirja [verkkojulkaisu]. Helsinki: Kustannus Oy Duodecim, päivitys 3.6.2016.

[D5] Punoittava tai vetistävä silmä (toimitus). Lääkärin tietokannat/Lääkärin käsikirja [verkkojulkaisu]. Helsinki: Kustannus Oy Duodecim, päivitys 12.9.2016.

[D6] Färkkilä M. Päänsärky. Lääkärin tietokannat/Lääkärin käsikirja [verkkojulkaisu]. Helsinki: Kustannus Oy Duodecim, päivitys 10.5.2016.

[D7] Kairaluoma M. Anaaliabsessi. Lääkärin tietokannat/Lääkärin käsikirja [verkkojulkaisu]. Helsinki: Kustannus Oy Duodecim, päivitys 2.8.2016.

[D8] Kairaluoma M. Peräpukamat. Lääkärin tietokannat/Lääkärin käsikirja [verkkojulkaisu]. Helsinki: Kustannus Oy Duodecim, päivitys 2.8.2016.

[D9] Kairaluoma M.  Anaalifissuura. Lääkärin tietokannat/Lääkärin käsikirja [verkkojulkaisu]. Helsinki: Kustannus Oy Duodecim, päivitys 2.8.2016.

[D10] Kairaluoma M.  Peräaukon kutina. Lääkärin tietokannat/Lääkärin käsikirja [verkkojulkaisu]. Helsinki: Kustannus Oy Duodecim, päivitys 2.8.2016.

[D11] Kairaluoma M. Verenvuotoa peräaukosta aiheuttavat taudit. Lääkärin tietokannat/Lääkärin käsikirja [verkkojulkaisu]. Helsinki: Kustannus Oy Duodecim, päivitys 2.8.2016.

[D12] Arkkila P. Kolonoskopia. Lääkärin tietokannat/Lääkärin käsikirja [verkkojulkaisu]. Helsinki: Kustannus Oy Duodecim, päivitys 11.2.2016.

[D13] Arkkila P. Aikuisen ummetus. Lääkärin tietokannat/Lääkärin käsikirja [verkkojulkaisu]. Helsinki: Kustannus Oy Duodecim, päivitys joulukuussa 2016.

[D14] Jalanko H. Korvatulehdus lapsella. Terveyskirjasto/Lääkärikirja Duodecim [verkkojulkaisu]. Helsinki: Kustannus Oy Duodecim, päivitys 6.12.2016.

[D15] Saarelma O. Korvavammat, korvalehden, korvakäytävän ja tärykalvon vammat. Terveyskirjasto/Lääkärikirja Duodecim [verkkojulkaisu]. Helsinki: Kustannus Oy Duodecim, päivitys 15.6.2016.

[D16] Atula T. Korvalehden vammojen hoito. Lääkärin tietokannat/Lääkärin käsikirja [verkkojulkaisu]. Helsinki: Kustannus Oy Duodecim, päivitys 16.8.2016.

[D17] Lumio J. Korvakäytävän tulehdus. Terveyskirjasto/Lääkärikirja Duodecim [verkkojulkaisu]. Helsinki: Kustannus Oy Duodecim, päivitys 30.9.2016.

[D18] Blomgren K. Vierasesine korvakäytävässä. Lääkärin tietokannat/Lääkärin käsikirja [verkkojulkaisu]. Helsinki: Kustannus Oy Duodecim, päivitys 14.5.2016.

[D19] Hirvonen T. Äkillinen kuulonmenetys. Lääkärin tietokannat/Lääkärin käsikirja [verkkojulkaisu]. Helsinki: Kustannus Oy Duodecim, päivitys 13.5.2016.

[D20] Atula T. Aikuisen korvanseudun kipu. Lääkärin tietokannat/Lääkärin käsikirja [verkkojulkaisu]. Helsinki: Kustannus Oy Duodecim, päivitys 16.8.2016.

[D21] Saarelma O. Korvakipu ja vuoto korvakäytävästä aikuisilla. Terveyskirjasto/Lääkärikirja Duodecim [online]. Helsinki: Kustannus Oy Duodecim, päivitys 1.7.2016.  Paakkari P. Kipulääkkeiden turvallinen käyttö. Terveyskirjasto/Lääkärikirja Duodecim [verkkojulkaisu]. Helsinki: Kustannus Oy Duodecim, päivitys 25.2.2016.

[D22] Lumio J. Korvakäytävän tulehdus. Terveyskirjasto/Lääkärikirja Duodecim [online]. Helsinki: Kustannus Oy Duodecim, päivitys 30.9.2016.

[D23] Hirvonen T. Äkillinen kuulonmenetys. Lääkärin tietokannat/Lääkärin käsikirja [online]. Helsinki: Kustannus Oy Duodecim, päivitys 13.5.2016.

[D24] Lääkärin tietokantojen toimitus. Alaselkäkipu. Lääkärin tietokannat/Lääkärin käsikirja [online]. Helsinki: Kustannus Oy Duodecim, päivitys 28.4.2017.

[D25] Saarelma O. Selkäkipu. Terveyskirjasto/Lääkärikirja Duodecim [online]. Helsinki: Kustannus Oy Duodecim, 24.5.2017.

[D26] Taimela S. Alaselän vaivat - liikuntaohje.  Terveyskirjasto/Lääkärikirja Duodecim [online]. Helsinki: Kustannus Oy Duodecim, 11.10.2016.

[D27] Paakkari P. Kipulääkkeiden turvallinen käyttö. Terveyskirjasto/Lääkärikirja Duodecim [online]. Helsinki: Kustannus Oy Duodecim, päivitys 25.2.2016.

[D28] Saarelma O. Vuodelepo ei ole vaikuttava hoitomuoto alaselkäkivussa tai iskiaksessa. Tutkimusnäyttö. Terveyskirjasto/Lääkärikirja Duodecim [online]. Helsinki: Kustannus Oy Duodecim, päivitys 10.3.2014.

[D29] Helenius I. Lapsen kipeä selkä. Lääkärin tietokannat/Lääkärin käsikirja [online]. Helsinki: Kustannus Oy Duodecim, päivitys 3.8.2016.

[D30] Atula T. Aikuisen korvanseudun kipu. Lääkärin tietokannat/Lääkärin käsikirja [online]. Helsinki: Kustannus Oy Duodecim, päivitys 16.8.2016.

[D31] Brander P. Hengenahdistus. Lääkärin tietokannat/Lääkärin käsikirja [online]. Helsinki: Kustannus Oy Duodecim, päivitys 18.5.2015.

[D32] Kervinen H. Akuutti sepelvaltimo-oireyhtymä ja sydäninfarkti. Lääkärin tietokannat/Lääkärin käsikirja [verkkojulkaisu]. Helsinki: Kustannus Oy Duodecim, päivitys 9.12.2016.

[D33] Voutilainen M. Refluksitauti.  Lääkärin tietokannat/Lääkärin käsikirja [verkkojulkaisu]. Helsinki: Kustannus Oy Duodecim, päivitys 7.6.2016.

[D34] Lääkärin käsikirja - Olkapään kiertäjäkalvosimen kiputilat [verkkojulkaisu] [http://www.terveysportti.fi/dtk/ltk/koti?p_artikkeli=ykt00463](http://www.terveysportti.fi/dtk/ltk/koti?p_artikkeli=ykt00463), päivitetty 11.5.2016

[D35] Lääkärin käsikirja - Jäätynyt olkanivel [verkkojulkaisu] [http://www.terveysportti.fi/dtk/ltk/koti?p_artikkeli=ykt01384](http://www.terveysportti.fi/dtk/ltk/koti?p_artikkeli=ykt01384), päivitetty 11.5.2016 Lääkärin käsikirja - Niska-hartiakipu [verkkojulkaisu] <http://www.terveysportti.fi/dtk/ltk/koti?p_artikkeli=ykt00459>, päivitetty 9.5.2016

[D36] Toimitus. Kipeä polvi.  Lääkärin tietokannat/Lääkärin käsikirja [verkkojulkaisu]. Helsinki: Kustannus Oy Duodecim, päivitys 25.8.2016.

[D37] Kantele A.  Mikrobien aiheuttamat ripulitaudit. Lääkärin tietokannat/Lääkärin käsikirja [verkkojulkaisu]. Helsinki: Kustannus Oy Duodecim, päivitys 15.7.2016.

[D38] Artikkelit "Virtastieinfektiot", "Virtsan perustutkimukset ja bakteeriviljely". Lääkärin käsikirja Kustannus Oy Duodecim  [verkkojulkaisu]

[D39] Lumio K ja Jalanko H. Sivuontelotulehdus (poskiontelotulehdus). Terveyskirjasto/Lääkärikirja Duodecim  [verkkojulkaisu]. Helsinki: Kustannus Oy Duodecim, päivitys 13.10.2014.

[D40] Haahtela T. Astman hoito. Terveyskirjasto/Lääkärikirja Duodecim  [verkkojulkaisu]. Helsinki: Kustannus Oy Duodecim, päivitys 11.5.2010.

[D41] Mustajoki P. Keuhkoahtaumatauti. Terveyskirjasto/Lääkärikirja Duodecim  [verkkojulkaisu]. Helsinki: Kustannus Oy Duodecim, päivitys 29.4.2014.

[D42] Brander P. Hengenahdistus. Lääkärin tietokannat/Lääkärin käsikirja  [verkkojulkaisu]. Helsinki: Kustannus Oy Duodecim, päivitys 18.5.2015.

[D43] Lääkärin tietokantojen toimitus. Aikuisen pitkittynyt yskä. Lääkärin tietokannat/Lääkärin käsikirja  [verkkojulkaisu]. Helsinki: Kustannus Oy Duodecim, päivitys 8.8.2016.

[D44] Mustajoki P. Yskä. Terveyskirjasto/Lääkärikirja Duodecim  [verkkojulkaisu]. Helsinki: Kustannus Oy Duodecim, päivitys 13.10.2014.

Preliminary studies investigating the likelihood of different combinations of symptoms and associated conditions requiring treatment [E1- E14]

[E1] Koskinen S, Lundqvist A, Ristiluoma N. Terveys, toimintakyky ja hyvinvointi Suomessa 2011. Terveyden ja hyvinvoinnin laitoksen julkaisuja, 2012. Saatavilla <http://www.julkari.fi/bitstream/handle/10024/90832/Rap068_2012_netti.pdf?sequence=1>

[E2] Norman DC. Fever in the Elderly. Clin Infect Dis. 2000;31(1):148-151. doi:10.1086/313896.

[E3] Arroll B, Kenealy T. Antibiotics for acute bronchitis. BMJ 2001;322:939 (doi: <http://dx.doi.org/10.1136/bmj.322.7292.939>).

[E4] McIsaac WJ, White D, Tannenbaum D, Low DE. A clinical score to reduce unnecessary antibiotic use in patients with sore throat. CMAJ 1998;158:75-83

[E5] Symptom Checkers: http://familydoctor.org/familydoctor/en/health-tools/search-by-symptom/cold-flu.html <http://www.mayoclinic.org/symptom-checker/select-symptom/itt-20009075>

[E6] McIsaac WJ, White D, Tannenbaum D, Low DE. A clinical score to reduce unnecessary antibiotic use in patients with sore throat. CMAJ 1998;158:75-83.

[E7] Bösner S ym. Heartburn or angina? Differentiating gastrointestinal disease in primary care patients presenting with chest pain: a cross sectional diagnostic study. Int Arch Med 2009;Dec 12;2:40.

[E8] British Medical Journal - Shoulder pain: diagnosis and management in primary care [verkkojulkaisu].  <http://www.ncbi.nlm.nih.gov/pmc/articles/PMC1283277/>, julkaistu 12.11.2005.

[E9] du Toit J, Hamilton W, Barraclough K. Risk in primary care of colorectal cancer from new onset rectal bleeding: 10 year prospective study. BMJ 2006;333:69-70.

[E10] Derry S, Wiffen PJ, Moore RA, Bendtsen L. Ibuprofen for acute treatment of episodic tension-type headache in adults. Cochrane Database of Systematic Reviews 2015, Issue 7. Art. No.: CD011474. DOI: 10.1002/14651858.CD011474.pub2.

[E11] Kallela M. Migreenin hoitoon yksilöllisiä vaihtoehtoja. Suomen Lääkärilehti 2017;71:3077-84.

[E12] Perry J, Stiell I, ym. Clinical decision rules to rule out subarachnoid hemorrhage for acute headache. JAMA. 2013;310:1248-55. doi: 10.1001/jama.2013.278018.

[E13] Stephens G, Derry S, Moore RA. Paracetamol (acetaminophen) for acute treatment of episodic tension-type headache in adults. Cochrane Database of Systematic Reviews 2016, Issue 6. Art. No.: CD011889. DOI: 10.1002/14651858.CD011889.pub2.

[E14] Veys L, Derry S, Moore RA. Ketoprofen for episodic tension-type headache in adults. Cochrane Database of Systematic Reviews 2016, Issue 9. Art. No.: CD012190. DOI: 10.1002/14651858.CD012190.pub2.
